# Supplementary figures and images for: Trend and burden of neural tube defects among cohort of pregnant women in Ethiopia: Where are we in the prevention and what is the way forward?
Source: PLoS One. 2022 Feb 18;17(2):e0264005. doi: 10.1371/journal.pone.0264005 (PMC8856542; doi:10.1371/journal.pone.0264005)

**Retrospective data collection tool (Check list)**


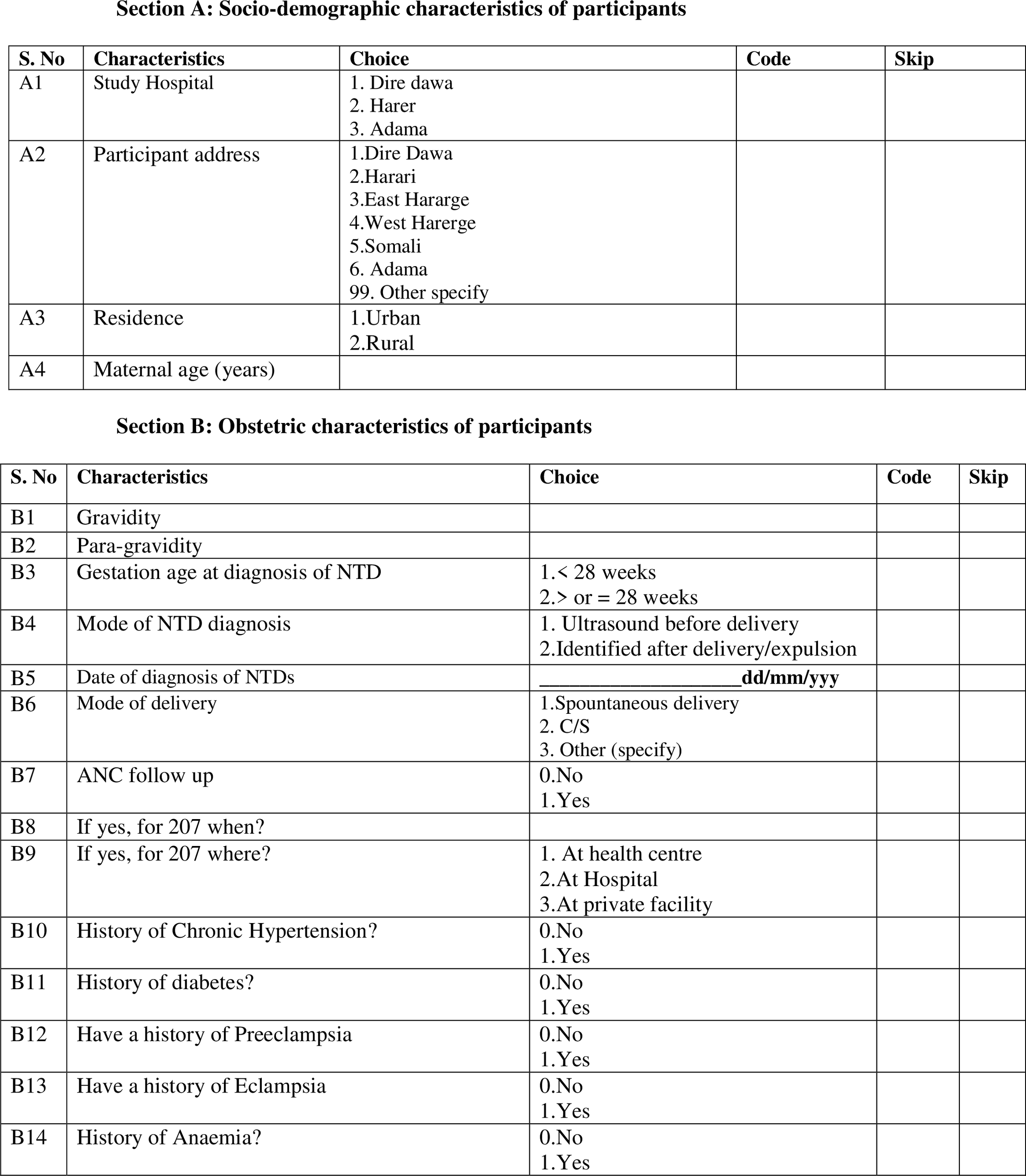


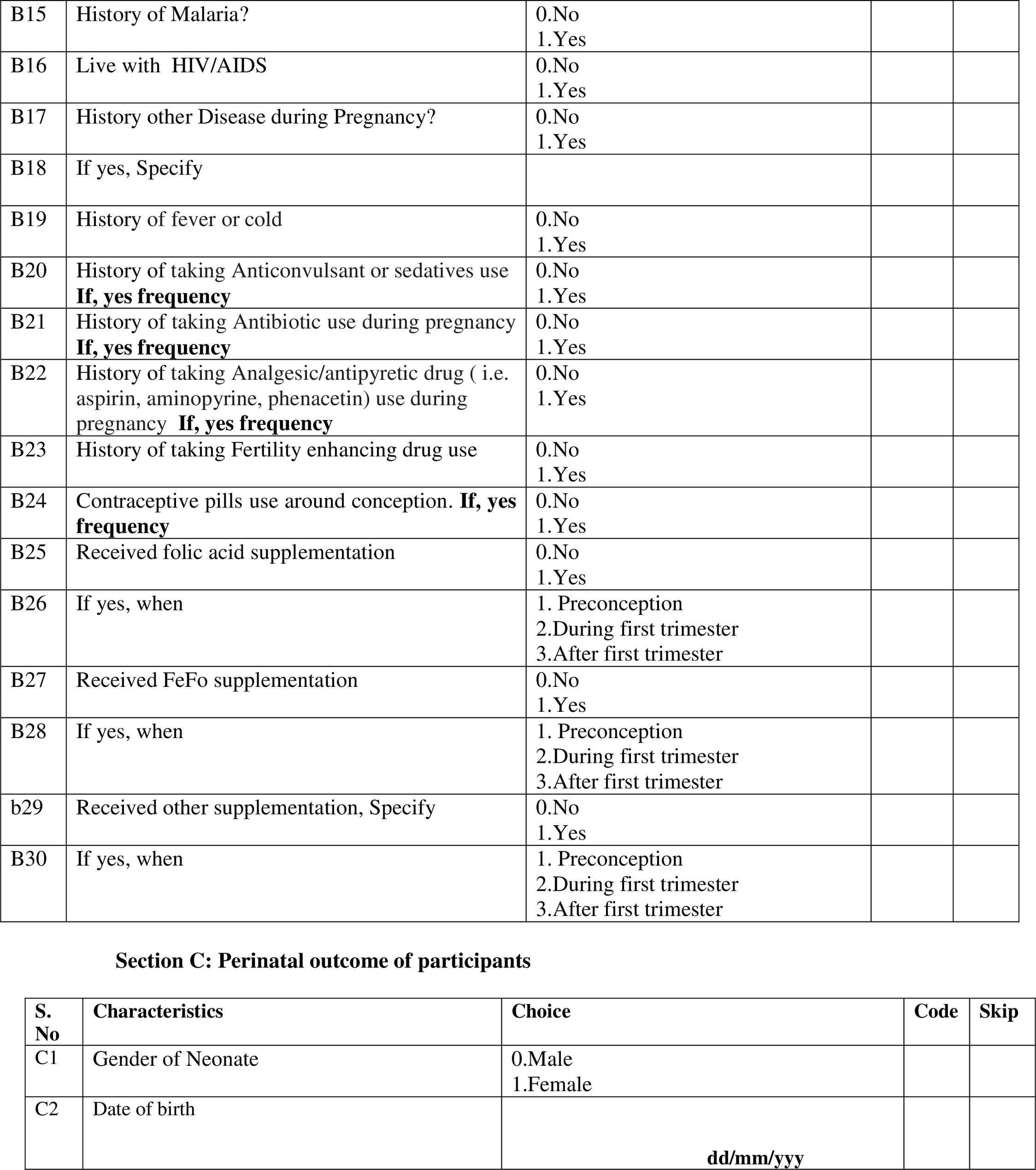


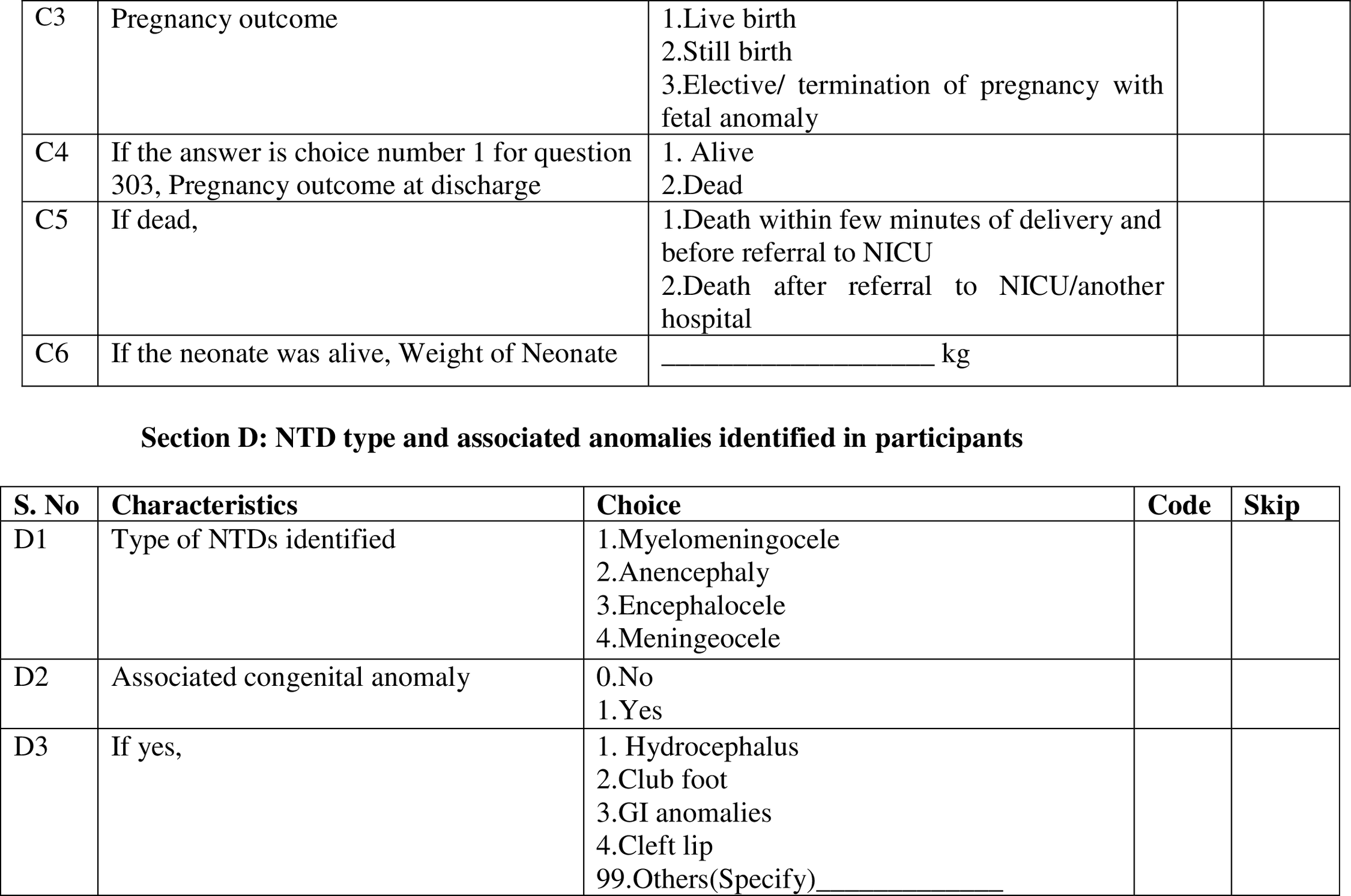

Supplement: S1 Checklist — (DOC) [file pone.0264005.s001.doc]
