# Supplementary material for: Trend and burden of neural tube defects among cohort of pregnant women in Ethiopia: Where are we in the prevention and what is the way forward?
Source: PLoS One. 2022 Feb 18;17(2):e0264005. doi: 10.1371/journal.pone.0264005 (PMC8856542; doi:10.1371/journal.pone.0264005)
Supplement: Attachment — Submitted filename: Response to Reviewer.doc [file pone.0264005.s002.doc]

**December 21, 2021**

**Editor-in-Chief of Plos one**

**Subject: Submitting the revised version of our Manuscript**

We are submitting the revised version our manuscript entitled “**Trend and Burden of Neural Tube Defects among cohort of pregnant women in Ethiopia: Where are we in the prevention and what is the way forward?**” for publication on Plos One. We have considered the reviewers’ comments with great appreciation and addressed them point by point as indicated in the following table. All authors have read the revised version of the manuscript and do not have any conflict of interest.

**Best regards**

**Anteneh Berhane**

**Corresponding author**

Point by Point responses to the reviewers; queries

| **Editor comment** | **Response** |
| --- | --- |
| 1. Title: the study area should be included in the title | Thank you. The comment is accepted and corrected  The corrected title based on comment is:  Trend and Burden of Neural Tube Defects among cohort of pregnant women in Ethiopia: Where are we in the prevention and what is the way forward? |
| 2. Background of the abstract doesn’t clearly show the existing burden of NTD in Ethiopia, and even elsewhere in the globe. Generally, burden of NTD should be numerically stated followed by the objectives showing the research gap the authors would like to address. | Thank you. The comment is accepted and corrected  Background  Neural tube defect is one of the top five most serious birth defects in the world. In Ethiopia an accurate estimate of the trend and burden of neural tube defects is still unknown. There hasn't been much research done on the prevalence and trend of neural tube defects in Eastern Ethiopia. To complement previous efforts of studies, the purpose of this study is to estimate the trend and burden of neural tube defects in Eastern Ethiopia as well as to investigate the epidemiological implications of the findings. |
| **3. Methods** of abstract should include sampling technique, measurement of NTD, type of data collection tool (adapted or adopted) and software for data entry and analysis. | Comment accepted and corrected  A facility-based retrospective cohort study was carried out from cohort pregnant women who delivered in selected hospitals. File records of all babies who were found to have neural tube defects could be reached between 2017 and 2019. A structured checklist was used to collect data. The incidence of each case was calculated by dividing the number of cases per year by the total number of live births in each hospital. To determine the linear trend of neural tube defects over time, linear trend of Extended Mantel-Haenszel chi-square was performed. Data were presented using frequencies and percentages. Data were anayzed using SPSS for windows version 25. |
| 4. In the last paragraph, it is better to include national incidence of NTD during the launch of different interventions in 2005 in Ethiopia | Thank you for your suggestion and we  Considered the suggestion and corrected it in the text of the document. |
| 5. Add a separate subsection of study area. Then, important details including nutrition culture and ANC follow up of the study population should be clearly stated so that any reader new to the Eastern society can get some understanding of the study area. | Comment accepted and Corrected. |
| 6. Why the authors considered only 2017-2019 time period which is actually not sufficient to show the time trend of NTD. | Because, we didn’t find the full recorded consistent document from the selective hospitals before 2017. Even if the existing data between 2017 and 2019 was not found fully, we have reported it as a limitation. |
| 7. Variables and operational definitions: Kindly include a separate subsection detailing measurement of the variables considered for the study | Comment accepted and Corrected.  Variables  Dependent Variable: Trend and burden of NTDs  Independent Variable: Socio-demographic, pregnancy, utilization of ANC, folic acid and IFA, maternal obstetric history, maternal health and drug history,  Operational definitions  NTDs cases: is defined as mothers who gave birth for a child with any type of NTDs (anencephaly, spina bifida, or encephalocele, or myelomeningocele or meningocele), irrespective of gestational age and live birth.  NTDs-affected pregnancy: can result in one of four outcomes: (1) an early fetal loss or miscarriage (defined as a spontaneous pregnancy loss at 20 completed weeks of gestation), (2) fetal death or stillbirth (defined as a spontaneous pregnancy loss at 20 completed weeks of gestation), (3) elective termination of pregnancy for fetal anomaly (eTOPFA), or (4) an affected live birth.  NTDs incidence was calculated as: = affected live births + affected still births + eTOPFAs for NTDs ×10,000  Live births |
| 8. Sampling technique: It would be more self -explanatory and easily understandable if the authors showed pictorial presentation (flow chart) of the sampling procedure including how many regions =>districts =>hospitals =>sample size (A cohort of 48,567 pregnant women delivered in three selected hospitals from 2017 to 2019) | Comment accepted and Corrected. But we believe that this figure presented in other way in results part. We suggest that may be it may be make redundancy |
| 9. Please upload your data collection tools (for both quantitative and qualitative) as additional file. | Comment accepted and uploaded. |
| 10. Ethical clearance: What beneficent actions will the authors provide the community in return for this study? | The purpose of this research is to investigate the new information that would be helpful to show the burden of NTDs in the study area and it is a baseline to take a possible intervention to reduce the neonate mortality, disability and mortality in the community by different actors. |
| 11. Please include a separate section that addresses incidence of NTD than mixing it with socio-demographic characteristics section. | Comment accepted and Corrected.  Changed to table |
| Discussion   12. The authors present severity of iron folate deficiency for the high burden of NTD in the study area than other regions. Why folate deficiency in Eastern Ethiopia is severe than other regions? Kindly give strong evidence, because it is clear that iron folate is uniformly distributed to all regions of the country. | Comment accepted.  Some of the probable reasons of inconsistency might be a different study subject, socio-demographic characteristics, locality, follow-up and reminding mechanism, knowledge of IFAS, and qualities of health services offered in the health facilities including counseling on its benefits, the average number of IFAS recommended during pregnancy, and belief on the use of IFAS. |
| **Reviewer 1**  1. In your method section, it is not clear that from how many hospitals in eastern Ethiopia, you was selected those three hospitals? | Thank you for your comment. There are more than 20 hospitals in the area but we only focused on the rank of hospital (tier of hospitals based on their service) that means referral, teaching hospitals and caseload because only this hospitals are only given the service regarding to NTDs case. Base on this there are 4 hospitals in that level and we took 3 hospitals. The reason why we left 1 hospital is due to a new upgraded and started after 2017. As we mention, our study started from 2017. |
| 2. I am not sure that your study design clearly retrospective cohort. What makes different from chart/record review? | As you know the main difference between the chart review and retrospective cohort is that chart review establish whether necessary information is available in the charts and inappropriate for study question. Retrospective studies may be based on chart reviews (data collection from the medical records of patients) and retrospective cohort studies (current or historical cohorts). So based on this facts we used the pretest structured questionnaire for data extraction from the mother and baby client card (including history of mothers) as well medical record book (about history of babies during delivery). |
| 3. Sample size determination and sampling procedure is not clear. | Thank you for your question and corrected in the manuscript. |
| 4. You should clearly describe morbidity/ major illnesses for current pregnancy and previous pregnancy separately. | Thank for your comment but as you know it is a secondary data and we did not found whether the illness was occurred previous pregnancy or current pregnancy. That is why we use the term “History of…” |
| 5. It will be better if you add risk factors for NTDs. | The risk factor is the next research area and as you read in this manuscript we suggested that it is better to investigate the determinant of developing NTDs in the area. |
| **Reviewer #2:**  1. Better to say a facility based instead of” institution based” | Thank you for your comment and corrected in manuscript. |
| 2. Why you want to focus from 2017 to 2019? What is new within this period? | As we mentioned in the limitation part we did not found the document before 2017. Even if from 2017 there are significant limitations to the recorded data. In some cases, the necessary investigation and complete history were not properly documented. Already we mention as a limitation of this study and recommended that it should be put the recorded data properly unless the exact figure of the incidence/burden not known which means we don’t know where we on the prevention of NTDs and we don’t have a data that NTDs contribution on neonate mortality. |
| 3. Are there only 3 hospitals in Eastern Ethiopia? | As we mentioned in the manuscript we select the hospitals based on case load and referral hospital. So based on this criteria we put there are 4 hospitals and we take 3 hospitals. The remained 1 hospital is due to a new upgraded and started after 2017. As we mention, our study started from 2017. |
| 4. Your study was” among cohort pregnant women who delivered in Dil-Chora, Hiwot Fana specialization teaching Hospital, and Adama Medical College Hospital” but the study population was medical records of cases who delivered or terminated or stillbirth or dead neonate with neural tube defects. Who were your exposed and unexposed groups? Please clarify this statement. | Yes, as you see in the document, data was retrieved from cohort pregnant women, who delivered or terminated with NTD affected pregnancy and history of women was also taken and analysed. We didn’t have a control group, so we are not classified as exposed and unexposed group. That is why we analysed the trend and burden of cases. |
| 5. “On the other hand, any type of NTDs case which is not clearly recorded and inconsistent data or data with more than 50% of values missing was excluded from the study” did the author excluded inconsistent data within the study period ? If yes how much? | Yes, 14 cases were excluded from the analysis. |
| 6. Was it closed cohort or open cohort study? | We believe that our study is closed cohort, because in closed cohort studies risk estimates are assessed in a short time interval as the ratio of cases over those at risk at the beginning of the study (incident proportion or risk in a given interval) and the cohort remains static during the study. So we assess the incidence/burden of NTDs in the study area. |
| 7. The author is required to clarify sample size calculation | Thank you for your comment and corrected in manuscript. |
| 8. In your sampling technique” all neural tube defects case those born in selected hospitals were included and selected conveniently” so what is the importance of talking about sample size calculation? | Thank you for your comment and corrected in manuscript. |
| 9. Who were your data collectors and supervisors? | Thank you for your comment and included in manuscript. |
